# Virus genomics and evolution: the transformative effect of new technologies and multidisciplinary collaboration on virus research and outbreak management

**DOI:** 10.1186/s13059-016-1019-8

**Published:** 2016-07-25

**Authors:** Camilla L. C. Ip, Oliver G. Pybus, Jennifer L. Gardy

**Affiliations:** 1 Wellcome Trust Centre for Human Genetics, University of Oxford, Oxford, OX37BN UK; 2 Department of Zoology, University of Oxford, Oxford, OX1 3PS UK; 3 School of Population and Public Health, University of British Columbia, Vancouver, BC V6T 1Z9 Canada; 4 British Columbia Centre for Disease Control, Vancouver, BC V5Z 4R4 Canada

## Abstract

The first Virus Genomics and Evolution Conference was held at the Wellcome Genome Campus in Hinxton, UK, 8–10 June 2016.

## Introduction

In recognition of the increasingly important role that genomics plays in virology and public health epidemiology, the Wellcome Genome Campus hosted the inaugural conference on Virus Genomics and Evolution in June 2016. This multidisciplinary conference brought together public health researchers who are generating viral genomic data from clinical samples and evolutionary biologists and bioinformaticians who are developing methods to interrogate these data.

The meeting highlighted the broad temporal and spatial scales over which viral genetic data are having an impact, from understanding the trajectory of resistance mutations during a single infection to understanding the spatial expansion of viral lineages during large epidemics. Several talks featured memorable accounts of the use of genomics in practical settings, from West African field hospitals to European courtrooms. Interestingly, there was little discussion of sequencing technologies themselves; rather the focus was on their practical, actionable applications.

In this report, we highlight how genomics is being used to explore viral origins and diversity and to understand transmission and pathogenesis. Additionally, we describe how real-time generation and analysis of viral genomic data are transforming outbreak surveillance and management.

## Virus discovery

Ian Lipkin (Columbia University, New York, USA) launched the meeting with tales of microbe-hunting across many species and diseases, emphasizing that detection of a virus genome in a sample is simply the first step in the process of linking an agent to disease. Viral discovery is fraught with false positives, and potential associations should be explored via multiple avenues, including serology and host response.

The scale of the viral discovery problem was highlighted by Eddie Holmes (University of Sydney, Australia), whose virus-hunting in diverse species—from unusual arthropods to sharks—shows that we have “barely scratched the surface of viral diversity”. Using metatranscriptomics, Holmes’ team demonstrated exceptional levels of virus diversity and revealed the dynamic nature of viral genome size and organization. A novel approach for viral discovery, using small interfering RNA (siRNA) as a marker of host response, was presented by Darren Obbard (University of Edinburgh, UK). Uncharacterized or underappreciated viral diversity is equally apparent at smaller taxonomic scales, as illustrated by the discovery of new viruses related to human hepatitis A virus (Jan Felix Drexler, University of Bonn Medical Centre, Germany) and by the re-emergence of human noroviruses from unsampled reservoir populations (Chris Ruis, University College London, UK).

## Public health diagnostics and surveillance

Perhaps the strongest theme of the meeting was the use of viral sequencing for surveillance and diagnostics in public health. The surge of interest in this area is no doubt due to the recent West African Ebola virus (EBOV) epidemic, which provided the backdrop for several talks. Kristian Anderson (The Scripps Research Institute, La Jolla, USA) explained how the Broad Institute’s Lassa Fever genomics infrastructure in Sierra Leone was quickly repurposed for Ebola virus sequencing and gave examples of epidemiological insights drawn from genomics during the outbreak. He cautioned that real-time genomics must lead to real-time action. Specifically, he reported that genomic data could have influenced public health conversations early in the outbreak; sequencing indicated that ongoing EBOV transmission was not due to multiple spillovers from an animal reservoir, yet messages about bushmeat consumption probably gave the opposite impression. The work presented by Kristian also demonstrated the utility of clinical metagenomics in an outbreak, revealing the associations between co-infection with malaria and Ebola mortality. Gustavo Palacios (US Army Medical Research Institute of Infectious Diseases (USAMRIID), Frederick, MD) described EBOV genomics in Liberia, where sequencing revealed the roles played by viral persistence in immune-privileged sites in transmission from survivors as the outbreak waned.

A glimpse of the future of surveillance was afforded by Gytis Dudas’ (Fred Hutchinson Cancer Research Center, Seattle, USA) meta-analysis of around 1600 EBOV genomes sequenced across the two-year outbreak. His phylogeographic analysis showed how EBOV lineages crossed from one country to another, avoided extinction by spreading within countries, and were magnified in urban areas. Access to a subset of these data during the outbreak resulted in border closures that limited the epidemic, demonstrating that genomic information, acquired in real time, could help to contain future epidemics. Ian Goodfellow (University of Cambridge, UK) set up an EBOV sequencing center in Sierra Leone and described how the attendant logistical challenges of such a project, from sourcing nitrogen canisters to managing samples, must not be underestimated. Ian demonstrated the benefits of sharing, whether it be of open-access data via platforms like virological.org and nextflu.org or of the sequencing equipment itself, which was eventually rehomed at the University of Makeni. Hinting at a subject that might feature at the next edition of the conference, Oliver Pybus (University of Oxford, UK) introduced the ZiBRA project, which is using portable nanopore sequencing in the field in Brazil to explore the dynamics of the Zika virus epidemic.

## From transmission to epidemics

The use of viral genomics as a tool to characterize transmission is not restricted to emerging epidemics, having been applied widely to established human pathogens. Examples presented at the meeting included reconstruction of a measles virus outbreak in Canada (Jennifer Gardy, University of British Columbia, Canada), community transmission of respiratory syncytial virus in Kenya (Charles Agoti, KEMRI-Wellcome Trust, Kenya), hospital outbreaks that were sequenced for infection control as part of the ICONIC (InfeCtion respONse through vIrus genomiCs) project (Paul Kellam, Sanger Institute, Hinxton, UK), and the use of HIV and hepatitis C virus (HCV) phylogenies to establish or disprove criminal transmission in court (Annemie Vandamme, KU Leuven, Belgium). Annemie explained why inferring the direction of transmission is not equivalent to inferring direct transmission and, through a series of real-world examples, she established the conditions that must be met to argue for transmission.

At a larger epidemic scale, Marion Koopmans (Erasmus Medical Centre, The Netherlands) discussed global norovirus dynamics as captured by the Noronet surveillance platform, including the recent emergence of recombinant strains that are undergoing rapid diversification and the association of excess mortality with new variants. Talks from Philippe Lemey (KU Leuven, Belgium) and Kirstyn Brunker (University of Glasgow, UK) demonstrated how other data sources can be merged with viral genomes to help us to understand epidemic dynamics, with examples showing how predictors such as spatial distance and case counts affected the Ebola outbreak, and how human-mediated connectivity and spatial heterogeneity influence rabies virus transmission in Tanzania.

## Antiviral treatment

The meeting also reflected a growing area of research in which virus genomics is used to guide the design of effective antiviral drug interventions. Despite the approval of new—but expensive—direct-acting antiviral drugs for HCV infection, we still do not know what determines whether treatment will be successful against the diverse range of HCV genotypes. As Ellie Barnes and M. Azim Ansari (University of Oxford, UK) explained, large-scale sequencing of both HCV and human genomes, followed by genome-wide association studies (GWAS), is beginning to tease apart the host and virus genetic components of treatment response.

Sequencing can also be used to observe the effects of drugs on viral genomes directly. Daniel Goldhill (Imperial College London, UK) showed evidence supporting the hypothesis that the experimental compound favipiravir acts to increase the mutation rate of influenza A viruses. Viral genomics is also being used to plan and evaluate large-scale treatment programs. In the final keynote address, Christophe Fraser (Imperial College London, UK) showed how viral phylogenetics is being used to identify the source of new HIV infections in the PopART community-randomized trial of HIV testing and treatment, which involves around 1.2 million people in sub-Saharan Africa. He described how the huge scale of such a project requires the development of new, efficient methods of evolutionary analysis, as well as rigorous thinking around sample size and power calculations for tests based on phylogenetic and molecular clock models.

## Evolutionary insights into pathogenesis

The importance of virus and host co-evolution was appreciated by the audience. Sunetra Gupta’s (University of Oxford, UK) dynamic models of the adaptive immune system indicated that decreasing levels of CD4+ T cells determined HIV viral loads during the chronic phase of infection, which, in turn, determined the speed of disease progression. Sarah Cobey (University of Chicago, USA) showed how influenza vaccines influence mutations in host B cells, and Andrea Tanzer (University of Vienna, Austria) asked whether, since Zika virus has been circulating for decades, there are any genetic changes that sparked the current outbreak in the Americas? By comparing the structure and folding kinetics of flaviviral RNA elements involved in host response and virulence, she identified specific mutations leading to RNA structural alterations and evidence for selective pressure by compensatory mutations.

## Concluding remarks

Although the conference focused on genomics and evolution—topics that might be considered more theoretical than applied—it was notable that many speakers talked as much about real people, some of whom have suffered or died from viral disease, as they did about data, theories, and technology. The frequency and scale of viral outbreaks in recent years has emphasized the urgency of research in virus genomics. Further, it has prompted efforts to shift academic culture towards closer collaborations between clinicians, biologists, mathematicians, and computer scientists and towards the real-time, open release and analysis of data, with the shared aim of securing the health of our world’s population. The next meeting is provisionally scheduled for June 2018.

## Abbreviations

EBOV, West African Ebola virus; HCV, hepatitis C virus

